# Application of Phenotyping Methods in Detection of Drought and Salinity Stress in Basil (*Ocimum basilicum* L.)

**DOI:** 10.3389/fpls.2021.629441

**Published:** 2021-02-18

**Authors:** Boris Lazarević, Zlatko Šatović, Ana Nimac, Monika Vidak, Jerko Gunjača, Olivera Politeo, Klaudija Carović-Stanko

**Affiliations:** ^1^Department of Plant Nutrition, Faculty of Agriculture, University of Zagreb, Zagreb, Croatia; ^2^Centre of Excellence for Biodiversity and Molecular Plant Breeding (CroP-BioDiv), Faculty of Agriculture, University of Zagreb, Zagreb, Croatia; ^3^Department of Seed Science and Technology, Faculty of Agriculture, University of Zagreb, Zagreb, Croatia; ^4^Department of Plant Breeding, Genetics and Biometrics, Faculty of Agriculture, University of Zagreb, Zagreb, Croatia; ^5^Department of Biochemistry, Faculty of Chemistry and Technology, University of Split, Split, Croatia

**Keywords:** high-throughput phenotyping, 3D scanning, chlorophyll fluorescence imaging, salinity stress, drought stress

## Abstract

Basil is one of the most widespread aromatic and medicinal plants, which is often grown in drought- and salinity-prone regions. Often co-occurrence of drought and salinity stresses in agroecosystems and similarities of symptoms which they cause on plants complicates the differentiation among them. Development of automated phenotyping techniques with integrative and simultaneous quantification of multiple morphological and physiological traits enables early detection and quantification of different stresses on a whole plant basis. In this study, we have used different phenotyping techniques including chlorophyll fluorescence imaging, multispectral imaging, and 3D multispectral scanning, aiming to quantify changes in basil phenotypic traits under early and prolonged drought and salinity stress and to determine traits which could differentiate among drought and salinity stressed basil plants. *Ocimum basilicum* “Genovese” was grown in a growth chamber under well-watered control [45–50% volumetric water content (VWC)], moderate salinity stress (100 mM NaCl), severe salinity stress (200 mM NaCl), moderate drought stress (25–30% VWC), and severe drought stress (15–20% VWC). Phenotypic traits were measured for 3 weeks in 7-day intervals. Automated phenotyping techniques were able to detect basil responses to early and prolonged salinity and drought stress. In addition, several phenotypic traits were able to differentiate among salinity and drought. At early stages, low anthocyanin index (ARI), chlorophyll index (CHI), and hue (HUE_2__*D*_), and higher reflectance in red (R_*Red*_), reflectance in green (R_*Green*_), and leaf inclination (LINC) indicated drought stress. At later stress stages, maximum fluorescence (F_*m*_), HUE_2__*D*_, normalized difference vegetation index (NDVI), and LINC contribute the most to the differentiation among drought and non-stressed as well as among drought and salinity stressed plants. ARI and electron transport rate (ETR) were best for differentiation of salinity stressed plants from non-stressed plants both at early and prolonged stress.

## Introduction

Basil (*Ocimum basilicum* L.) is the most important species from the genus *Ocimum* of the subfamily Nepetoideae under the family Lamiaceae (Chowdhury et al., 2017). It is a well-known medicinal, aromatic, and ornamental plant (Radácsi et al., 2010; Purushothaman et al., 2018). Because it is exceptionally rich in essential oils, it is commonly produced for economic purposes (Caliskan et al., 2017). It has a wide range of applications; it is used as a spice in many cuisines, as an ingredient for commercial fragrances, flavors, to improve the food products shelf life, in traditional medicine and phytotherapy (Labra et al., 2004; Carović-Stanko et al., 2010; Radácsi et al., 2010; Mishra et al., 2012; Carović-Stanko et al., 2013; Purushothaman et al., 2018).

Although it is cultivated worldwide, traditionally main basil-producing areas are related to the Mediterranean region. Global climate scenarios classified the Mediterranean as one of the most prone regions to climate change (Intergovernmental Panel on Climate Change [IPCC], 2013). Moreover, in the Mediterranean region, climate change has already caused a significant increase in frequency and intensity of drought events (Vicente-Serrano et al., 2014) and along with demographic pressures has led to increased land desertification and salinization (Safriel, 2009). Like most cultivated plants, basil is shown to be sensitive to different abiotic stresses such as drought (Kordi et al., 2013; Damalas, 2019) and salinity (Attia et al., 2011). Drought stress and salinity share many features since both decrease water availability for the roots and cause osmotic stress to plants (Munns, 2002; Flexas et al., 2004). Osmotic stress caused by drought, salinity, or both impacts many morphological traits and physiological processes in basil, such as relative growth rate, water relations, transpiration rate, water use efficiency, nutrient uptake, stomatal conductivity, photosynthesis, senescence, yield, and yield components (Radácsi et al., 2010; Attia et al., 2011; Osakabe et al., 2014; Caliskan et al., 2017; Negrão et al., 2017). Besides osmotic stress which occurs in the early phase of salinity stress, prolonged salinity causes ionic stress, mainly concerning Na^+^ and Cl^–^ accumulation (Munns, 2002). The deleterious effect of both drought and salinity depends on the timing of occurrence, intensity, and duration of a stressful factor (Munns, 2002; Tuberosa, 2012); however, if the stress is prolonged, plant growth and productivity are severely reduced (Osakabe et al., 2014). Therefore, it is essential to define plants’ morphophysiological status in the early stages of drought and salinity stress before plants are severely damaged.

Various plant phenotyping platforms have recently been developed, aiming to detect the physiological status of plants exposed to stressful conditions. Most widely used methods for non-destructive studying of plant phenotypic traits under stressful conditions are chlorophyll fluorescence imaging (Brestic and Zivcak, 2013; Bresson et al., 2015; Humplík et al., 2015; Awlia et al., 2016; Yao et al., 2018), multispectral imaging (Huang et al., 2015; Wang et al., 2018), and 3D multispectral scanning (Vadez et al., 2015; Paulus, 2019). Employing those techniques gives valuable insights into plant performance under specific environmental conditions and estimates different traits such as light utilization by photosystem II (PSII) and underlying biochemical processes, leaf pigment content, leaf chemical composition, leaf and shoot morphological and architectural traits, etc.

Due to often co-occurrence of drought and salinity, similar effects (symptoms) which they cause on plants, and the complexity of morphophysiological plant adaptations to drought and salinity, it is difficult to differentiate between traits related to these two stresses. In this study, we included integrative approach which combines whole-plant chlorophyll fluorescence imaging, multispectral imaging, and 3D multispectral scanning, aiming to:

1. Quantify changes in basil phenotypic traits under drought and salinity stress;
2. Determine the most responsive phenotypic trait (traits) to drought and salinity stress;
3. Assess potential differences in trait expression between drought and salinity stressed basil plants.

## Materials and Methods

### Plant Material and Growth Conditions

Seeds of *O. basilicum* “Genovese” (MAP02282) were obtained from the Collection of Medicinal and Aromatic Plants held at the Department of Seed Science and Technology, Faculty of Agriculture, University of Zagreb, Croatia. To get enough plant material, seeds were sown in germination trays (containers). Germination and initial plant growth were conducted in the greenhouse with a mean daily temperature of 22.5°C. Fifteen days after germination, seedlings were transplanted into 2 L plastic pots filled with 600 g of potting substrate Substrat 1 (Klasmann-Deilmann GmbH) with the addition of 5 g of NPK 15-15-15 per pot. Transplanted plants were transferred to growth chamber under 25/20°C, 16/8 h day/night regime, 70% relative air humidity, and 250 μmol m^–2^ s^–1^ of photosynthetic photon flux density (PPFD) provided by Valoya L35, NS12 spectrum LED lights (Valoya Oy, Helsinki Finland), and grown for 10 days allowing plants to adjust to the chamber conditions.

### Experimental Setup and Treatments

After 10 days of adaptation, 50 uniformly developed plants were selected for the experiment, and all measurements (Table 1) were performed to determine the initial state. After initial measurements, treatments were applied. The treatments included well-watered control (treatment C), moderate salinity stress (S1), severe salinity stress (S2), moderate drought stress (D1), and severe drought stress (D2). Control plants (C) were regularly irrigated with distilled water to keep the substrate’s volumetric water content (VWC) between 45 and 50%. Plants under moderate salinity stress (S1) were irrigated twice a week with 150 mL of 100 mM NaCl solution and those under severe salinity stress (S2) with 150 mL of 200 mM NaCl solution. Soil VWC of plants under both salinity stress levels was kept at 45–50%. The VWC of plants under moderate drought (D1) was between 25 and 30%, whereas those under severe drought stress (D2) was kept between 15 and 20%. Soil VWC and electrical conductivity (EC) were measured daily with Theta probe ML2x sensor connected to the HH2 moisture meter (Delta-T Devices Ltd., Cambridge, United Kingdom) substrate-specific calibration. Measurements were performed by inserting three pins of the sensor perpendicular to the substrate level at three different points of each pot, and the average value was calculated (Supplementary Figures SF1a,b).

**TABLE 1:** List of all measured traits with the abbreviations and measuring device.

**No.**	**Abbr.**	**Trait**	**Trait type**	**Device**
1	DB	Digital biomass	Morphological	PlantEye
2	PH	Plant height (mm)		
3	TLA	Total leaf area (cm^2^)		
4	LAI	Leaf area index		
5	LAP	Leaf area projected (cm^2^)		
6	LINC	Leaf inclination		
7	LANG	Leaf angle (°)		
8	LPD	Light penetration depth (mm)		
9	R_Red_	Red reflectance	Color and multispectral	CropReporter
10	R_Green_	Green reflectance		
11	R_Blue_	Blue reflectance		
12	R_FarRed_	Far-red reflectance		
13	R_NIR_	Near-infrared reflectance		
14	HUE_2__D_	Hue 2D		
15	SAT	Saturation		
16	VAL	Value		
17	HUE_3__D_	Hue 3D		PlantEye
18	CHI	Chlorophyll index	Vegetation indices	CropReporter
19	ARI	Anthocyanin index		
20	GI	Greenness index		PlantEye
21	NDVI	Normalized difference vegetation index		
22	NPCI	Normalized pigments chlorophyll ratio index		
23	PSRI	Plant senescence reflectance index		
24	F_0_	Minimum chlorophyll fluorescence	Chlorophyll fluorescence	CropReporter
25	F_m_	Maximum chlorophyll fluorescence		
26	F_v_/F_*m*_	The maximum quantum yield of PSII		
27	F_s’_	Steady-state fluorescence yield		
28	F_m’_	Maximum chlorophyll fluorescence		
29	F_q’_/F_m’_	The effective quantum yield of PSII		
30	ETR	Electron transport rate		
31	NPQ	Non-photochemical quenching		

The experiment was set up as a completely randomized design (CRD) with the 10 plants per treatment. Plants were grown for 4 weeks until the onset of flowering. All the measurements were performed at the initial state (time T0) and after first (T1), second (T2), and third (T3) week.

### Plant Measurements

All measured plant traits along with abbreviations and the device used for measuring are shown in Table 1.

#### 3D Multispectral Scanning

Plants were scanned using a PlantEye F500 multispectral 3D scanner (Phenospex, Heerlen, Netherlands). PlantEye measures the spectral reflectance in Red (peak wavelength 620–645 nm), Green (peak wavelength 530–540 nm), Blue (peak wavelength 460–485 nm), Near-Infrared (peak wavelength 820–850 nm), and the 3D laser (940 nm) of the plant. Resolution of the PlantEye was set up as follows: Z-range (the distance measured from the scanner down) 40 cm, Y-resolution (Vscan = 50 mm s^–1^) 1 mm, X-resolution 0.19 mm, and Z-resolution < 0.1 mm. Calculation of vegetation indices and morphological parameters starts from the 3D point cloud from which the 3D plant model is built by integrated Phena software (Phenospex, Heerlen, Netherlands). All points that belong to the same sector are triangulated. Triangles were created by connecting adjacent points. Different vegetation indices and morphological parameters were calculated using HortControl software (Phenospex, Heerlen, Netherlands).

#### Vegetation Indices

Calculated vegetation indices from 3D plant model were: HUE_3__*D*_, calculated in the same way as described above, but using 3D plant model, Greenness index (GI) (2 × R_*Green*_ – R_*Red*_ – R_*Blue*_)/(R_*Green*_ + R_*Red*_ + R_*Blue*_), normalized difference vegetation index (NDVI) (R_*NIR*_ – R_*Red*_)/(R_*NIR*_ + R_*Red*_) (Rouse et al., 1974), normalized pigments chlorophyll ratio index (NPCI) (R_*Red*_ – R_*Blue*_)/(R_*Red*_ + R_*Blue*_) (Peñuelas et al., 1995), and plant senescence reflectance index (PSRI) PSRI = (R_*Red*_ - R_*Green*_)/(R_*NIR*_) (Merzlyak et al., 1999).

#### Morphological Parameters

Calculated morphological parameters from 3D plant model were: plant height (PH; mm) calculated as distribution of elementary triangles along the *z*-axis; leaf area projected (LAP; cm^2^) calculated as an area of the projection of all elementary triangles on *X*–*Y* plane; total leaf area (TLA; cm^2^) calculated as the sum of all triangle domains, where each domain represents a group of triangles that form a uniform surface; digital biomass (DB; cm^3^) calculated as the product of the height and 3D leaf area; leaf area index (LAI, mm^2^ mm^–2^) calculated as TLA/sector size; leaf inclination (LINC; mm^2^ mm^–2^) which describes how leaves on the plant are erected and calculated as TLA/LAP; leaf angle [LANG; degree (°)]; and light penetration depth (LPD; mm) measured by the deepest point in which the laser can penetrate the canopy along the *z*-axis.

#### Chlorophyll Fluorescence and Multispectral Imaging

Chlorophyll fluorescence and multispectral imaging were performed using the CropReporter^TM^ (PhenoVation B.V., Wageningen, Netherlands). The CropReporter^TM^ consists of a cabinet with a camera system that houses controller computer, charge-coupled device (CCD) camera with optical filter wheel and focusing unit, integrated high-intensity red light-emitting diodes (LEDs) for excitation of the photosynthesis, LEDs at six spectral bands [broadband white (3000 K), far-red (730 nm), red (660 nm), green (520 nm), blue (460 nm), and UV/blue (405 nm)], controllable in intensity (0–780 μmol m^–2^ s^–1^), and spectrum for spectral imaging. All images are captured with the same lens (10 Mp lens, 200 Lp mm^–1^ resolution, 400–1000 nm spectral range) and CCD camera (1.3 Mp, 1296 × 966 pixels), with real 14-bit signal resolution. Plants were imaged at 80 cm distance from the camera. The output is 16-bit RAW format, and automatic analysis of chlorophyll fluorescence, color, and multispectral images was performed by DA^TM^ software (PhenoVation B.V., Wageningen, Netherlands).

#### Chlorophyll Fluorescence Imaging

Plants were imaged with the optimized quenching protocol or dark-to-light slow fluorescence induction (Brestic and Zivcak, 2013), which includes dark adaptation, measurement of the induction curve of the dark-adapted plant followed by actinic light switching on for light adaptation, and measurement of induction curve of light-adapted plants.

For chlorophyll fluorescence measurements of dark-adapted plants (overnight dark adaptation), saturating light pulse (4500 μmol m^–2^ s^–1^ for an 800 ms) was used. Minimum chlorophyll fluorescence (F_0_) was measured after 20 μs, and maximum chlorophyll fluorescence (F_*m*_) was measured after saturation. Four dark frames were captured and averaged to one single frame during the time red LEDs were off; 20 frames were captured for the induction curve during 800 ms; integration time for capturing the chlorophyll fluorescence images was 200 μs.

Following the measurement of dark-adapted plants, plants were relaxed in the dark for 15 s, and then actinic lights (300 μmol m^–2^ s^–1^) were switched on enabling plants to adapt to light for 5 min. Steady-state fluorescence yield (F_*s’*_) was measured at the onset of the saturating pulse, and maximum chlorophyll fluorescence (F_*m’*_) of light-adapted plants was measured at saturation, using the saturating pulse intensity (4500 μmol m^–2^ s^–1^). Again, four dark frames were captured and averaged to one single frame during the time red LEDs were off; 20 frames were captured for the induction curve during 800 ms; integration time for capturing the chlorophyll fluorescence images was 200 μs.

Measured F_0_, F_*m*_, F_*m’*_, and F_*s’*_ were used for calculation of the following fluorescence parameters:

The maximum quantum yield of PSII (F_*v*_/F_*m*_): F_*v*_/F_*m*_ = (F_*m*_ − F_0_)/F_*m*_ (Genty et al., 1989)
Effective quantum yield of PSII (F_*q’*_/F_*m’*_): F_*q’*_/F_*m’*_ = (F_*m’*_ − F_*s’*_)/F_*m’*_ (Genty et al., 1989)
Electron transport rate (ETR) = F_*q’*_/F_*m’*_ × PPFD × (0.5) (Genty et al., 1989)
Non-photochemical quenching (NPQ) = (F_*m*_ − F_*m’*_)/F_*m’*_ (Bilger and Björkman, 1990).

#### 2D Multispectral Imaging

After chlorophyll fluorescence imaging, color and spectral reflectance (R) images were captured at 300 μmol m^–2^ s^–1^ produced by broadband white LEDs. Reflectance images were captured at R_*Red*_—640 nm, R_*Green*_—550 nm, R_*Blue*_—475 nm, R_*Chlorophyll*_ (R_*Chl*_)—730 nm, R_*Anthocyanin*_ (R_*Anth*_)—540 nm, R_*NIR*_—769 nm, and R_*FarRed*_—710 nm. During imaging, spectral ratio (R_*A*__*nth*_: R_*FarRed*_: R_*NIR*_) and color ratio (R_*Red*_: R_*Green*_: R_*Blue*_) were kept constant.

From reflectance images, chlorophyll index (CHI) and anthocyanin index (ARI) were calculated using the following equations: CHI = (R_*Chl*_)^–1^ − (R_*NIR*_)^–1^ (Gitelson et al., 2003), and ARI = (R_*Anth*_)^–1^ − (R_*FarRed*_)^–1^ (Gitelson et al., 2001). Hue, saturation, and value were calculated after converting R_*Red*_, R_*Green*_, and R_*Blue*_ into values between 0 and 1.

Hue (0–360°) was calculated as follows:

HUE = 60 × [0 + (R_*Green*_ − R_*Blue*_)/(max − min)], if max = R_*Red*_;
HUE = 60 × [2 + (R_*Blue*_ − R_*Red*_)/(max − min)], if max = R_*Green*_;
HUE = 60 × [4 + (R_*Red*_ − R_*Green*_)/(max − min)], if max = R_*Blue*_.
360 was added in case of HUE < 0.

Value (0–1) was calculated as: VAL = (max + min)/2, while max and min were selected from the R_*Red*_, R_*Green*_, R_*Blue*_. Saturation (0–1) was calculated as: SAT = (max – min)/(max + min) if VAL > 0.5, or SAT = (max – min)/(2.0 – max – min) if VAL < 0.5, while max and min were selected from the R_*Red*_, R_*Green*_, R_*Blue*_.

### Statistical Analysis

The analysis of variance (ANOVA) with repeated measures was performed using the MIXED procedure in SAS 9.4 (SAS Institute Inc, 2011, Cary, NC, United States) as described by Littell et al. (2000). The model included the effects of treatment (control, C; moderate salinity stress, S1; severe salinity stress, S2; moderate drought stress, D1; severe drought stress, D2), time (T0–T4; used for repeated measures), and treatment × time interaction on 31 variables (described above and given in Table 1). The optimal covariance structure model was chosen based on Akaike information criterion with a correction for small sample sizes (AICc). Examined covariance matrix structure types included unstructured (UN), variance components (VC), compound symmetric (CS), first-order autoregressive [AR(1)], and Toeplitz (TOEP) (Supplementary Table ST1). Tukey’s honest significant difference *post hoc* test was performed for partitioned F-tests (SLICE option) to examine the significance of treatments differences within time and time differences within treatments. Pearson’s correlation coefficients were calculated and tested using the CORR procedure in SAS 9.4. A principal component analysis (PCA) was performed on 31 variables, including all data points (five treatments × four times of measurements × 10 plants = 200) using the PRINCOMP procedure in SAS 9.4. The first two principal components were used to construct a biplot. Discriminant analyses (DAs) were performed using STEPDISC, DISCRIM, and CANDISC procedures in SAS 9.4. A stepwise DA (STEPDISC) was used to select a subset of variables for use in discriminating among the treatments at each time (T1, T2, T3). The significance level for adding variables in the forward selection mode, or removing them in the backward elimination mode, was *P* ≤ 0.15. The chosen subset of variables was evaluated for the performance as the discriminant criterion (DISCRIM) for correct classification of plants into their respective treatments by estimating misclassification probabilities with cross-validation. A canonical DA (CANDISC) was performed based on the minimal set of variables that differentiated best between treatments, and the first two canonical variables (CVs) were plotted. The same procedure was performed at T1 using only the control treatment (C) and the two salinity stress levels (S1 and S2). The obtained discriminant function was finally applied to the total dataset (including two drought stress levels, D1 and D2).

## Results

**FIGURE 1:**
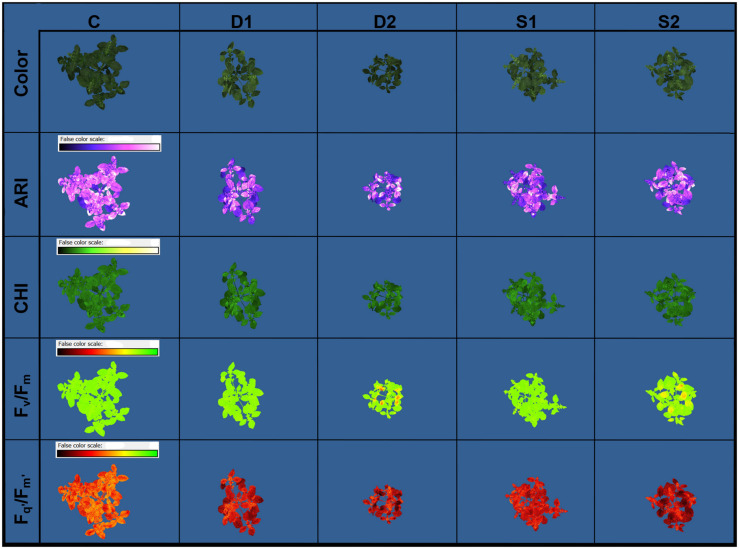
Basil color and pseudo-color images of anthocyanin index (ARI), chlorophyll index (CHI), maximum quantum yield of PSII (F_*v*_/F_*m*_), and the effective quantum yield of PSII (F_*q’*_/F_*m’*_) under control (C), moderate drought stress (D1), severe drought stress (D2), moderate salinity stress (S1), and severe salinity stress (S2), captured by CropReporter at second measurement time (T2; 14 days after onset of treatments).

Novel plant phenotyping techniques, including chlorophyll fluorescence imaging, multispectral imaging, and 3D multispectral scanning, were employed to investigate basil physiological and morphological responses to drought and salinity stress. Phenotypic traits (Table 1) were evaluated at the onset (T0) of stressful treatments and three time points 7 (T1), 14 (T2), and 21 (T3) days after the onset of treatments. Soil VWC and EC were daily monitored, and differences among treatments in VWC and EC were obtained at T1 (Supplementary Figure SF1). Color and pseudo-color images showing the effect of drought and salinity stress on several selected traits (RGB, F_*v*_/F_*m*_, F_*q’*_/F_*m’*_, CHI, and ARI) are presented in Figure 1.

Analysis of variance table and means for all measured traits with the *post hoc* test results are provided as Supplementary Tables ST2, ST3.

### Effect of Drought and Salinity Stress on Morphological Parameters

**FIGURE 2:**
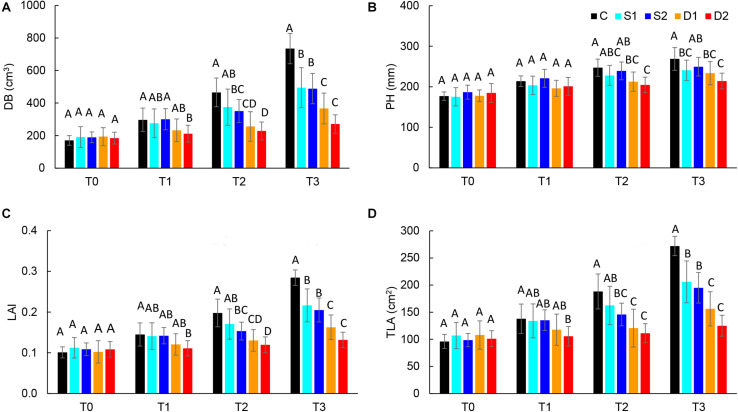
Selected 3D morphological traits: **(A)** digital biomass (DB), **(B)** plant height (PH), **(C)** leaf area index (LAI), and **(D)** total leaf area (TLA), of basil plants grown under control (C), moderate drought stress (D1), severe drought stress (D2), moderate salinity stress (S1), and severe salinity stress (S2) measured at 0 (T0), 7 (T1), 14 (T2), and 21 (T3) days after the beginning of treatments. *Post hoc* comparisons of the means were performed using Tukey’s HSD test at *P* < 0.05; different letters indicate significant differences among treatments within each measurement time.

For the quantification of morphological changes under drought and salinity stress, plants were scanned with a 3D multispectral scanner. All morphological traits including PH, LAP, TLA, LAI, DB, LINC, LANG, and LPD were automatically calculated from 3D plant models. The selected morphological traits are presented in Figure 2, while all the results are given in Supplementary Table ST3.

Both drought and salinity stress affected all measured morphological traits except LPD (Supplementary Table ST2). The earliest (observed at T1) and most remarkable changes were caused by severe drought stress and were related to the decrease in leaf area (TLA, LAP, and LAI) and DB. Similar morphological changes (decrease in DB, TLA, LAP, and LAI) were found in severe salinity and moderate drought only after the prolonged time (T2 and T3) (Figures 2A,C,D and Supplementary Table ST3). Significantly lower DB, TLA, LAI, and LAP found at T3 in plants from both moderate and severe drought treatments compared to plants from salinity treatments indicate that drought has a more profound effect on plant morphology than salinity.

### Effect of Drought and Salinity Stress on Visible and Multispectral Reflectance

Both CropReporter and PlantEye are designed and calibrated for the multispectral measurements; thus, masking or subtracting the background signals is integrated within software reflectance calculations, and there is no need for additional calibration of absolute reflection values. Thus, we present absolute reflectance parameters, i.e., reflectance in red (R_*Red*_), green (R_*Green*_), blue (R_*Blue*_), near-infrared (R_*NIR*_), and far-red (R_*FarRed*_). In addition to visible reflectance as an alternative for color analysis, we have used hue (HUE), saturation (SAT), and value (VAL). HUE considers all three measured colors (red, green, and blue), but it is represented as one channel arranged in a rainbow colors chart with values 0–360°. The saturation (SAT) of each color represents its intensity (pale or intense color), and the value (VAL) shows if the color is dark or bright.

The color analysis results show that reflectance in red, green, and blue significantly increased in severe drought and severe salinity treatment, whereas moderate drought and moderate salinity did not affect R_*Red*_, R_*Green*_, and R_*Blue*_ (Supplementary Table ST3). Hence, severe drought treatment caused the earliest changes in color reflectance. Namely, severe drought significantly increased R_*Red*_, R_*Green*_, and R_*Blue*_ already at T1, whereas severe salinity increased R_*Blue*_ at T1, R_*Red*_ at T2, and R_*Green*_ at T3 (Supplementary Table ST3). HUE_2__*D*_ was more sensitive to drought and less sensitive to salinity than color reflectance parameters. Namely, a significant decrease in HUE_2__*D*_ was obtained from T1 for both moderate and severe drought treatment, and at last measurement (T3) for moderate and severe salinity treatments (Supplementary Table ST3). Hence, R_*NIR*_ decreased from T2 for all stressful treatments; however, the most pronounced decrease was found for severe drought treatment.

**FIGURE 3:**
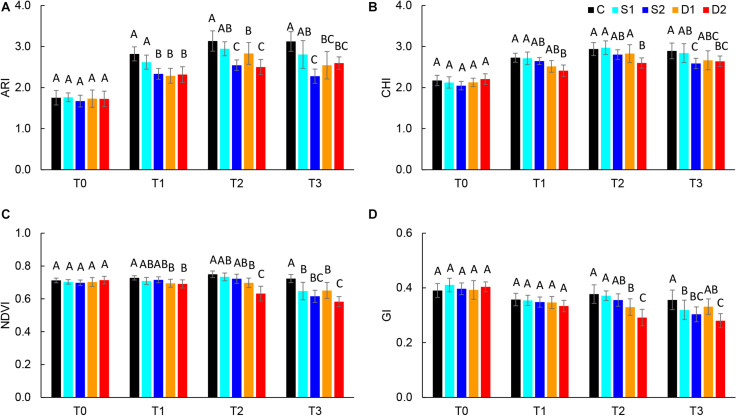
Selected vegetation indices: **(A)** anthocyanin index (ARI), **(B)** chlorophyll index (CHI), **(C)** normalized digital vegetation index (NDVI), and **(D)** greenness index (GI) of basil plants grown under control (C), moderate drought stress (D1), severe drought stress (D2), moderate salinity stress (S1), and severe salinity stress (S2) measured at 0 (T0), 7 (T1), 14 (T2), and 21 (T3) days after the beginning of treatments. *Post hoc* comparisons of the means were performed using Tukey’s HSD test at *P* < 0.05; different letters indicate significant differences among treatments within each measurement time.

Absolute reflectance values were used for the calculation of different vegetation indices: ARI, CHI, GI, NDVI, NPCI, and PSRI. Results of selected vegetation indices are presented in Figure 3, and all results are given in Supplementary Table ST3. Compared to salinity, most studied vegetation indices were earlier affected by drought treatments. Namely, severe drought treatment significantly decreased ARI, CHI, and NDVI already at T1, GI at T2, and increased PSRI at T3, whereas moderate drought decreased ARI and NDVI at T1 and GI at T2 (Figures 3A–C). On the other hand, the earliest change caused by severe salinity was decreased ARI observed at T1(Figure 3A). Other indices (NDVI, GI, and CHI) were affected by salinity treatments only after the prolonged time (T3) (Figures 3B–D).

### Effect of Drought and Salinity Stress on Chlorophyll Fluorescence Parameters

The effect of drought and salinity stress on basil photosynthetic performance was assessed by measuring chlorophyll fluorescence parameters [the maximum quantum yield of PSII (F_*v*_/F_*m*_), the effective quantum yield of PSII (F_*q’*_/F_*m’*_), ETR, and non-photochemical quenching (NPQ)].

**FIGURE 4:**
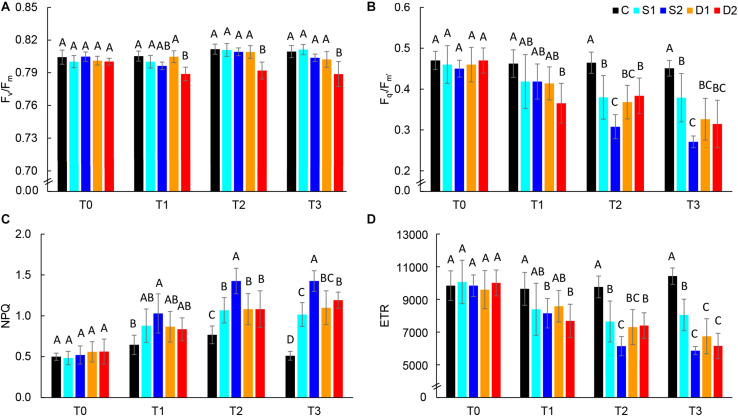
Selected chlorophyll fluorescence parameters: **(A)** maximum quantum yield of PSII (F_*v*_/F_*m*_), **(B)** effective quantum yield of PSII (F_*q’*_/F_*m’*_), **(C)** non-photochemical quenching (NPQ), and **(D)** electron transport rate (ETR) of basil plants grown under control (C), moderate drought stress (D1), severe drought stress (D2) moderate salinity stress (S1), and severe salinity stress (S2) measured at 0 (T0), 7 (T1), 14 (T2), and 21 (T3) days after the beginning of treatments. *Post hoc* comparisons of the means were performed using Tukey’s HSD test at *P* < 0.05; different letters indicate significant differences among treatments within each measurement time.

Chlorophyll fluorescence parameters are presented in Figure 4 and Supplementary Table ST3. At the first measurement time (T0), there were no differences among treatments in all measured chlorophyll fluorescence parameters indicating uniformity of selected plants for the experiment. At T0, the average F_*v*_/F_*m*_ value was 0.80, and average F_*q*__’_/F_*m’*_ was 0.46, indicating non-stressed plants. Hence, in the control plants, chlorophyll fluorescence parameters were relatively consistent over measurements (T0–T3) (Supplementary Table ST3). Earliest changes (observed at T1) were found for severe drought treatment which caused a significant decrease in F_*v*_/F_*m*_, F_*q*__’_/F_*m’*_, and ETR and severe salinity treatment which caused a significant increase in NPQ and decrease in ETR (Figure 4). Hence, after prolonged stress (T2 and T3) F_*q*__’_/F_*m’*_, ETR and NPQ were most affected by severe salinity treatment, although these traits were also affected by all stressful treatments (Figures 4B–D). On the other hand, F_*v*_/F_*m*_ was only affected by severe drought treatment (T3) (Figure 4A), indicating insensitivity of this parameter to moderate drought and salinity stress.

### Correlation and Differentiation Among Drought and Salinity Affected Traits

**FIGURE 5:**
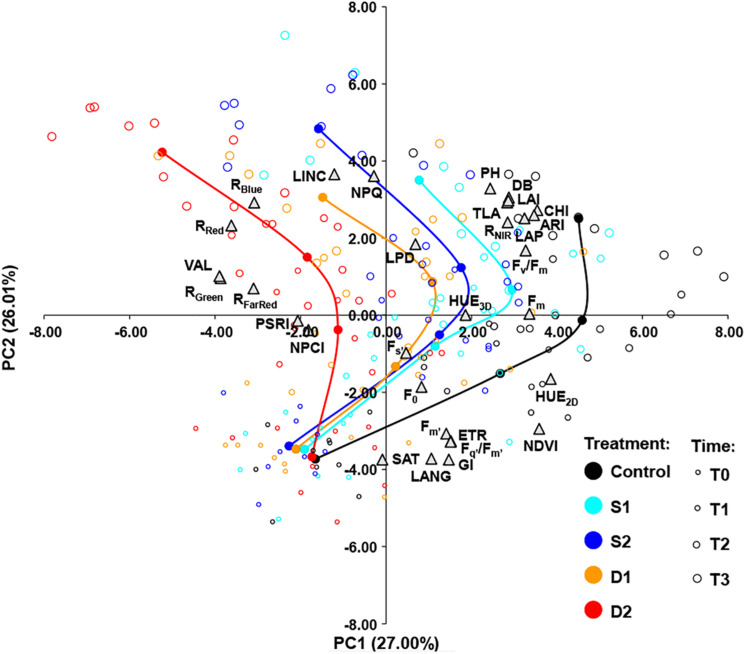
Biplot of principal component analysis (PCA) based on 31 traits (gray triangles) of 200 basil samples from five treatments [open circles: control, C (black); moderate salinity stress, S1 (cyan); severe salinity stress, S2 (blue); moderate drought stress, D1 (orange); severe drought stress, D2 (red)] in four-time points (T0–T3) depicted by the size of the circles. Filled circles (connected by a line) represent the barycenters of treatments in each time point.

The relationships among measured traits across treatments (C, D1, D2, S1, and S2) and measurement times (T0, T1, T2, and T3) were investigated by using Pearson’s correlation coefficients (Supplementary Table ST4) and PCA (Figure 5). A strong positive correlation was found among several groups of traits. Namely, among morphological traits (DB, PH, LAI, LAP, and TLA), among light-adapted chlorophyll fluorescence traits (F_*m’*,_ F_*q*__’_/F_*m’*_, ETR), among vegetation indices, and multispectral reflectance traits (NDVI, HUE_2__*D*_, and GI, CHI, ARI, and R_*NIR*_) and color reflectance traits (R_*Red*_, R_*Green*_, R_*Blue*_, R_*FarRed*_, and VAL). Traits that were positively correlated with NDVI and CHI tended to correlate negatively with traits from the group of R_*Red*_, R_*Green*_, R_*Blue*_, VAL, and R_*FarRed*_ (Supplementary Table ST4).

The principal component biplot was constructed by the first two axes, which account for 53% of the total variance. In general, the first principal component (PC1) differentiated among treatments and the second principal component (PC2) differentiated among measurement time. Also, the *treatment* × *measurement time* interaction could be noticed as the differences among treatments became more pronounced over time (Figure 5). PC1 most strongly positively correlated (≥0.70) with HUE_2__*D*_, NDVI, CHI, and ARI and negatively (≤−0.70) with R_*Red*_, R_*Green*_, and VAL. The PC2 correlated most strongly (≥0.70) with LINC and NPQ, and negatively (≤−0.70) with GI, LANG, and SAT (Figure 5).

The STEPDISC was performed at each measurement time (T1, T2, and T3) to assess traits that discriminate best among the treatments in the early and later stages of drought and salinity stress. Out of 31 traits, 15 were chosen to be the best differentiating factors among treatments at T1, 11 traits at T2, and 17 at T3 (Supplementary Table ST5). A subset of variables was evaluated for the performance as the DISCRIM for correct classification of plants into their respective treatments by estimating misclassification probabilities with cross-validation. Results of cross-validation are given as Supplementary Tables ST5a–ST5d and show that discriminant function correctly classified 90–100% plants into their respective treatments.

**FIGURE 6:**
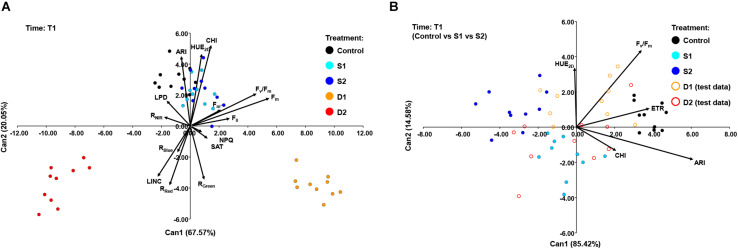
Biplot of the canonical discriminant analysis (CDA) at **(A)** time 1 (T1) based on 15 variables (shown as vectors) that best discriminate among five treatments [control, C (black); moderate salinity stress, S1 (cyan); severe salinity stress, S2 (blue); moderate drought stress, D1 (orange); severe drought stress, D2 (red)] and **(B)** at time 1 (T1) based on five variables (shown as vectors) that best discriminate among three treatments [full circles: control, C (black); moderate salinity stress, S1 (cyan); severe salinity stress, S2 (blue)]. The discriminant function was also applied to treatments D1 and D2 treated as test data [open circles: moderate drought stress, D1 (orange); severe drought stress, D2 (red)].

A CANDISC was performed based on the minimal set of variables that differentiated best between treatments, and the first two CVs were plotted. At T1, CANDISC based on 15 traits showed that the first two CVs explained 67.57 and 20.05% of the variation among treatments, respectively (Figure 6A). The first CV (CV1) discriminated between moderate drought (D1) and severe drought treatment (D2) and was strongly correlated with F_*m*_ (0.73) and F_*v*_/F_*m*_ (0.61). The second CV (CV2) discriminated between drought stress treatments (D1 and D2) from the control (C) and both salinity treatments (S1 and S2) and was correlated with CHI (0.70), HUE_2__*D*_ (0.62), and ARI (0.60) (Figure 6A). The additional procedure was performed using only the control treatment (C) and the two salinity stress levels (S1 and S2) which enabled discrimination among salinity stress and control in the early phase of the stress (T1). The obtained discriminant function was finally applied to the complete dataset (including two drought stress levels, D1 and D2) (Figure 6B). The CV1 discriminated between control (C) and salinity treatments (S1 and S2), explaining 85.42% of the total variance, and was strongly correlated with ARI (0.81), ETR (0.510), and F_*v*_/F_*m*_ (0.46). Whereas the second (CV2) discriminated between salinity treatments (S1 and S2) but accounted for only 14.58% of the total variance. CV2 was correlated with F_*v*_/F_*m*_ (0.58) and HUE_2__*D*_ (0.45) (Figure 6B).

**FIGURE 7:**
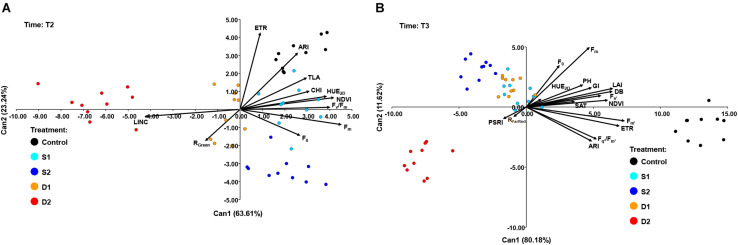
Biplot of the canonical discriminant analysis (CDA): **(A)** at time 2 (T2) based on 11 variables (shown as vectors) and **(B)** at time 3 (T3) based on 17 variables (shown as vectors) that best discriminate among five treatments [control, C (black); moderate salinity stress, S1 (cyan); severe salinity stress, S2 (blue); moderate drought stress, D1 (orange); severe drought stress, D2 (red)].

At T2, CANDISC based on 11 traits showed that the first two CVs explained 63.61 and 23.24% of the variation, respectively (Figure 7A). The CV1 was positively correlated with F_*m*_ (0.91), F_*v*_/F_*m*_ (0.81), HUE_2__*D*_ (0.78), and NDVI (0.84), whereas the CV2 was correlated with ARI (0.86) and ETR (0.63). CV1 discriminated between drought stress treatments (D1 and D2) from control (C) and both salinity treatments (S1 and S2), while CV2 discriminated between control (C) and severe salinity (S2) (Figure 7A).

At the prolonged time (T3), CANDISC based on 17 traits showed that the first two CVs explained 80.18 and 11.62% of the variation, respectively (Figure 7B). The CV1 was positively correlated with F_*s’*_ (0.70), F_*m’*_ (0.92), F_*q’*_/F_*m’*_ (0.66), ETR (0.87), ARI (0.61), DB (0.81), GI (0.61), TLA (0.81), LAI (0.81), and PH (0.77), whereas the second (CV2) was correlated with F_*m*_ (0.63). CV1 discriminated between control and stress treatments (D1, D2, S1, and S2), whereas CV2 discriminated between severe drought (D2) and other stress treatments (D1, S1, and S2) (Figure 7B).

## Discussion

Drought and salinity are considered as the two most common constraints in crop production, whose frequency and severity are estimated to increase in the future (Munns, 2002; Flexas et al., 2004; Munns and Tester, 2008; Tuberosa, 2012). In addition, drought-prone arid and semiarid regions, such as the Mediterranean basin, are often subjected to high salinity levels in soil or irrigation water. The early stage of salinity stress (osmotic phase) causes similar symptoms to those which occur under drought (Munns, 2002; Flexas et al., 2004). The recent development of automated phenotyping techniques enabled integrative and simultaneous quantification of multiple morphological and physiological traits under different stressful conditions.

Morphological parameters calculated from 3D plant models showed that both drought and salinity affected basil morphology and reduced growth. Most affected were traits related to leaf area and DB. Decreased biomass and reduced leaf area are commonly described morphological changes under drought and salinity conditions (Munns, 2002; Flexas et al., 2004; Munns and Tester, 2008), and are related to adjustments in heat dissipation and transpiration under stressful conditions (Bridge et al., 2013). Earlier and more profound reduction of morphological traits was found for drought stress treatments compared to salinity treatments. Growth reduction under salinity stress is related to osmotic pressure changes (soil water potential) (Munns, 2002; Munns and Tester, 2008). Awlia et al. (2016) found a reduction in *Arabidopsis* rosette area 7 days after the beginning of treatments with 100 and 150 mM NaCl solution. Thus, observed earlier reduction in DB leaf related traits under severe drought treatment compared to salinity treatments could indicate that during the first 7 days salinity treatments did not prevent water uptake to the same extent as was the case in severe drought treatment. However, similarly to our findings, Paul et al. (2019) found a higher reduction in biomass and leaf area under drought compared to salinity stressed wheat cultivars. Moreover, other measured traits (chlorophyll fluorescence parameters) indicate that salinity, as well as drought, caused significant stress to the basil plants already after 1 week.

Both drought and salinity increased reflectance in red, green, and blue and decreased NIR reflectance with the earliest and more pronounced changes obtained on plants from severe drought treatment. Similar changes in visible and NIR reflectance are often described under stressful conditions (Peñuelas and Filella, 1998; Mulla, 2013; Li et al., 2014). High NIR reflectance is generally related to healthy plants (Peñuelas and Filella, 1998; Li et al., 2014). However, NIR reflectance is not affected by leaf pigment content; instead, it is determined by leaf optical properties related to leaf morphology, thickness, water content, and light scattering (Merzlyak et al., 2003). This is supported in our research by the obtained positive correlation between NIR reflectance and morphological traits, and their simultaneous reduction in stress treatments. On the other hand, an increase in red and blue reflectance and decrease in HUE_2__*D*_ indicate the decrease in absorptance of the photosynthetic active radiation, probably caused by decreased chlorophyll content under stress treatments. A simultaneous decrease in GI and NDVI and increased PSRI support this statement. GI and NDVI are related to chlorophyll content and green biomass (Peñuelas and Filella, 1998; Merzlyak et al., 2003; Mulla, 2013; Li et al., 2014) whereas PSRI represents chlorophyll to carotenoid ratio and is related to leaf senescence (Merzlyak et al., 2003). In addition, the obtained increase in green reflectance under salinity stress was in line with the results reported by Awlia et al. (2016). These authors hypothesized that the anthocyanin accumulation in stressed leaves causes plants’ darker green color under salinity stress. However, in our experiment, this was not the case because the ARI of both salinity and drought-stressed plants decreased compared to control. Thus, higher green reflectance or darker leaves could be related to the accumulation of colored products of polyphenol oxidation which also occurs under different stressful conditions (Merzlyak et al., 2003). Decreased ARI under both salinity and drought is not in line with anthocyanins’ role as the photoprotective pigments, whose concentration increases under stressful conditions (Gitelson et al., 2001). Although anthocyanins are also abundant in juvenile plant organs and senescing leaves (Gitelson et al., 2001), their accumulation in senescing leaves enables nutrient remobilization (Feild et al., 2001). Thus, observed more substantial ARI increase in control plants than plants from stress treatments could be a species-specific trait or be related to higher growth rates in control plants.

Chlorophyll fluorescence imaging revealed that both drought and salinity stress had a more substantial effect on NPQ, ETR, and F_*q’*_/F_*m’*_ compared to F_*v*_/F_*m*_ which was affected only by severe drought. Although F_*v*_/F_*m*_ is the most frequently used parameter for estimation plant PSII performance under stressful conditions (Baker, 2008; Brestic and Zivcak, 2013), many authors reported that it is not sensitive to early or moderate water stress (Bukhov and Carpentier, 2004; Massacci et al., 2008) or salinity stress (Baker and Rosenqvist, 2004; Moradi and Ismail, 2007; Awlia et al., 2016). Bresson et al. (2015) found that *Arabidopsis thaliana* plants retain stable F_*v*_/F_*m*_ values over a prolonged period of drought stress; however, plants show bimodal F_*v*_/F_*m*_ distribution with regions showing high and low F_*v*_/F_*m*_, respectively. On the other hand, the observed increase in NPQ and a concomitant decrease in F_*q’*_/F_*m’*_ and ETR are in line with previous reports on drought (Yao et al., 2018) and salinity (Awlia et al., 2016). An increase in NPQ indicates activation of the leaf’s photoprotective processes (Maxwell and Johnson, 2000), whereas a decrease in F_*q’*_/F_*m’*_ and ETR is related to stomatal closure and CO_2_ limitation (Brestic and Zivcak, 2013). Besides, at prolonged stress, the NPQ is higher, and ETR is lower in severe salinity than in drought stress, indicating the ion toxicity caused by prolonged exposition to salinity stress. It could also indicate possible different photoprotective pathways such as the xanthophyll cycle, lutein cycle, and photorespiration in plants under drought and salinity stress. This assumption is supported by the fact that salinity stress affects more genes in comparison with drought (Chaves et al., 2009).

To assess the traits which are most responsive to drought and salinity, PCA was performed. PC1 corresponds to the differences between control and stress treatments, and PC2 corresponds to differences between measurement time. According to PC1 highest values of HUE_2__*D*_, NDVI, CHI, and ARI are related to control plants, whereas the highest values of R_*Red*_, R_*Green*_, and VAL are related to plants from stress treatments. Furthermore, these differences were more pronounced at the latter stages of stress.

The DA was performed at each measurement time to identify which traits differentiate best among salinity, drought, and control treatments at early and prolonged stress. At the early phase (T1), CV1 discriminated between moderate and severe drought and was strongly correlated with F_*m*_ and F_*v*_/F_*m*_. Although F_*v*_/F_*m*_ was a relatively stable trait, the fact that at T1 it only decreased in severe drought enabled it to differentiates between moderate and severe drought treatment. The CV2 discriminated drought stress treatments (D1 and D2) from other treatments (C, S1, and S2) and was strongly correlated with CHI, HUE_2__*D*_, and ARI. Due to the strong effect of drought treatments, the CANDISC did not differentiate between salinity stress and control treatment at T1. Thus, the additional procedure was performed using only control (C) and the two salinity stress levels (S1 and S2). The obtained discriminant function was finally applied to the total dataset (including two drought stress levels). This analysis shows that CV1 discriminated between control and salinity treatments and was strongly correlated with ARI and ETR, indicating that the earliest response to salinity stress is a decrease in ARI and ETR. At the latter stage (T2), CV1 discriminated between drought stress treatments and other treatments and was positively correlated with F_*m*_, F_*v*_/F_*m*_, HUE_2__*D*_, and NDVI. The CV2 discriminated between control and severe salinity, again based on ARI and ETR. After prolonged stress (T3), CV1 discriminated between control and all stress treatments and was correlated with F_*s’*_, F_*m’*,_ F_*q’*_/F_*m’*,_ ETR, ARI GI, TLA, LAI, and PH, indicating that prolonged salinity and drought stress cause similar phenotypic changes on basil plants.

## Conclusion

This study has shown that automated phenotyping techniques with simultaneous quantification of multiple morphological and physiological traits can detect plant response to early and prolonged salinity and drought stress. Moreover, several phenotypic traits were able to differentiate among salinity and drought stress. At early stages, low ARI, CHI, HUE_2__*D*_, higher LINC, and higher reflectance in red and green indicated drought stress and thus differentiated it from non-stressed and salinity stressed plants. At later stages of stress maximal fluorescence in dark-adapted state (F_*m*_), HUE_2__*D*_, NDVI, and LINC contribute the most to the differentiation among drought and non-stressed, as well as among drought and salinity stressed plants. Due to its insensitivity to moderate drought and salinity stress, F_*v*_/F_*m*_ differentiated between severe and moderate drought stress in early phases. ARI and ETR were best for differentiation of salinity stressed plants from non-stressed plants both at early and prolonged time.

Differences obtained in chlorophyll fluorescence parameters indicate that photosynthetic performance is differently affected by drought and salinity. Thus, combining used phenotyping techniques with gas exchange measurements, thermal imaging, and quantification of stomatal properties would give a comprehensive insight into the physiological background of basil responses to drought and salinity. Besides, using selected traits could serve to identify tolerant and sensitive genotypes in breeding programs and timely detection of salinity and drought stress in the field.

## Data Availability Statement

The original contributions presented in the study are included in the article/Supplementary Material. Further inquiries can be directed to the corresponding author/s.

## Author Contributions

BL and KC-S designed the experiment. KC-S provided basil seeds. ZŠ and JG analyzed the data. ZŠ, KC-S, and BL interpreted the results. OP, AN, and MV conducted the experiments, performed measurements, and contributed to writing the introduction and materials and methods. BL wrote the manuscript. ZŠ and KC-S made revisions. All authors contributed to the article and approved the submitted version.

## Conflict of Interest

The authors declare that the research was conducted in the absence of any commercial or financial relationships that could be construed as a potential conflict of interest.

## Supplementary Material

Supplementary Figure 1 Average readings of soil **(A)** volumetric water content (VWC) and **(B)** electroconductivity (EC). Three measurements per pot were recorded and averaged once per day, through the whole experimental period. T0, T1, T2, and T3 denote time points at which phenotyping measurement was performed; 0, 7, 14, and 21 days after the onset of treatments, respectively.

Supplementary Table 1 The choice of the optimal model of covariance structure used in the analysis of variance (ANOVA) with repeated measures.

Supplementary Table 2 Analysis of variance (ANOVA) for measured phenotypic traits of basil grown in different treatments: control (C), moderate salinity stress (S1), severe salinity stress (S2), moderate drought (D1), and severe drought (D2). Measurements were performed at four different times: 0 (T0), 7 (T1), 14 (T2), and 21 (T3) days after the onset of treatments. **(a)** ANOVA for repeated measures. **(b)** ANOVA test with SLICE option to examine the significance of each treatment across time. **(c)** ANOVA test with SLICE option to examine the significance of treatments within time.

Supplementary Table 3 The least-square means for partitioned F-tests (SLICE option) **(a)** to examine the significance of treatments across time and **(b)** to examine the significance of treatments within time.

Supplementary Table 4 Correlation matrix among studied phenotypic traits of basil grown in control (C), moderate salinity stress (S1), severe salinity stress (S2), moderate drought (D1), and severe drought (D2). Measurements were performed at four different times: 0 (T0), 7 (T1), 14 (T2), and 21 (T3) days after the onset of treatments.

Supplementary Table 5 Discriminant analysis results. Chosen traits are shown and the results of cross-validation for the classification of a chosen subset of variables into their respective groups/treatments [control (C), moderate salinity stress (S1), severe salinity stress (S2), moderate drought (D1), severe drought (D2)] at **(a)** T1 (7 days), **(b)** T1 for the C, S1 and S2, **(c)** T2 (14 days), and **(d)** T3 (21 days).
