# Supplementary figures and images for: Application of Phenotyping Methods in Detection of Drought and Salinity Stress in Basil (Ocimum basilicum L.)
Source: Front Plant Sci. 2021 Feb 18;12:629441. doi: 10.3389/fpls.2021.629441 (PMC7929983; doi:10.3389/fpls.2021.629441)

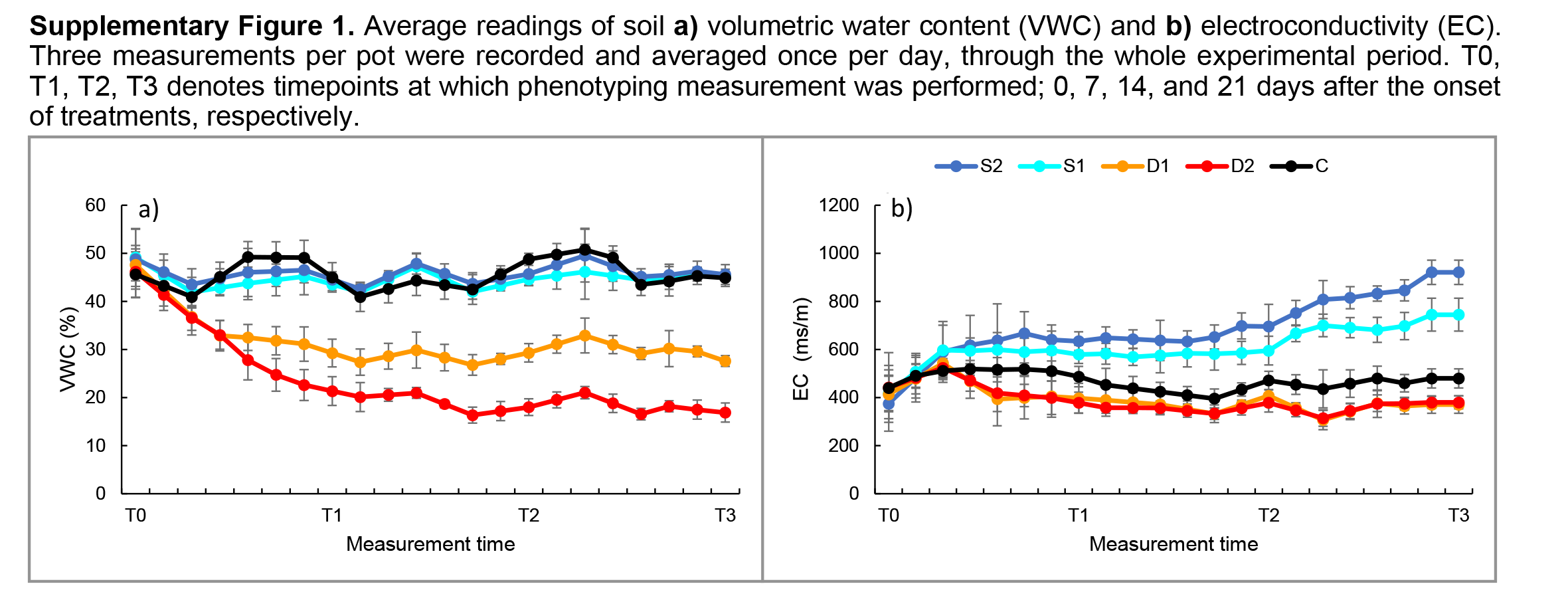

Supplement: Supplementary Figure 1 — Average readings of soil (A) volumetric water content (VWC) and (B) electroconductivity (EC). Three measurements per pot were recorded and averaged once per day, through the whole experimental period. T0, T1, T2, and T3 denote time points at which phenotyping measurement was performed; 0, 7, 14, and 21 days after the onset of treatments, respectively. [file Image_1.TIF]
